# 2022 Janet Doe Lecture, health science libraries in the emerging digital information era: charting the course

**DOI:** 10.5195/jmla.2023.1626

**Published:** 2023-04-21

**Authors:** Michael Kronenfeld

**Affiliations:** 1 mkronenf@yahoo.com, University Librarian Emeritus, A. T. Still Memorial Library, A.T. Still University of the Health Sciences, Kirksville, MO.

## Abstract

The great challenge medical library professionals are facing is how we evolve and respond to the emerging digital era. If we successfully understand and adapt to the emerging digital information environment, medical librarians/Health Information Professionals (HIPs) can play an even greater role in the advance in the health care of our nation and its residents. The opportunities and challenges are at the level we successfully responded to in the late 1960's and the 1970's under the leadership of the National Library of Medicine with its MEDLARS/Medline programs and Medical Library Assistance Act which enabled medical libraries to enter what I have referred to as *The Golden Age of Medical Libraries.* In this presentation, I focused on the transition of the health-related print Knowledge-Based Information base to the emerging digital health-related ecosystem. I review how this transition is being driven by evolving information technology. The development of “data driven health care” built on this emerging information ecosystem is being led by the National Library of Medicine's 2017-2027 Strategic plan and the Medical Library Association's programs in support of developing medical librarian/HIP's training, skills, and services to support their users access and use of this rapidly expanding health information ecosystem. I then present a brief description of the digital health information ecosystem that is just starting to emerge and the emerging new roles and services HIPs and their libraries are developing to support effective institutional access and use.

I am honored to have been selected to present this year's Janet Doe Lecture.

## FOUNDING MOTHERS OF MLA

Significant medical libraries in this country were primarily started and led by physicians such as John Shaw Billings and William Osler in the half century after the Civil War. Lay library staff, mostly women, served as the vocational worker bees who actually ran the libraries.

In the late 1930s and after WWII, a group of women consciously turned medical librarianship into a profession and were our profession's ‘Founding Mothers'. These included Marcia Noyes, Mary Louise Marshall, Estelle Broadman, Eileen Cunningham, and Janet Doe. In researching and writing my history of our profession I learned about their important roles in the profession I was so fortunate enough to work in for almost 45 years, making me especially pleased to have been selected to present this year's Janet Doe Lecture.

## FRONT COVER OF THE HISTORY BOOK

This lecture grew out of the writing of my recently published history of our profession titled “A History of Medical Libraries and Medical Librarianship: From John Shaw Billings to the Digital Era.” Today I will focus on our transition from the print-based medical library to the digitally based health information support ecosystem and from medical librarians to Health Information Professionals (HIPs).

In preparing and presenting my Doe Lecture I have been facing a major challenge: How in an hour to present on a macro or strategic level the great challenge our profession is facing as we evolve and respond to the emerging digital era. How we, as a profession, respond to this challenge will greatly impact the future of our profession. If we successfully understand and adapt to the emerging digital information environment opportunities as medical librarians/HIPs we can play an even greater role in the advance in the health care of our nation and its residents.

## MEDLARS, NNLM

I believe this opportunity and challenge is at the level we successfully responded to in the late 1960's and the 1970's under the leadership of the National Library of Medicine with its MEDLARS/Medline programs and Medical Library Assistance Act which enabled medical libraries to enter what, in our history, we referred to as “The Golden Age of Medical Libraries.”

I want to start with the basic concept that underlies our profession and my lecture which is that the basic or principle objective of medical libraries and medical librarians/HIPs is to improve the access and use of knowledge based information/information/data to improve our nation's health – both in the public health and clinical care areas. In the paper era, libraries, as the local repositories of KBI, were the most effective way of accomplishing this but they were the tool in support of achieving the library's mission.

In researching and writing our profession's history I discovered that one reason for our success has been our ability to adapt and evolve as the health care and information environments have advanced. With the emergence of the digital information environment we are again faced with the challenge and opportunity in our transformation from medical librarians facilitating access and use of the print based Knowledge-Based Information (KBI) collections provided by our libraries to Health Information Professionals (HIPs) working collaboratively with the units and staff we support facilitating the effective access to, use, and management of digitally based, increasingly accessible KBI/Information/data. My challenge to you – the current and future practitioners and leaders – is to understand and respond to the opportunities and challenges this transition is presenting.

I am starting my presentation with a brief review of the era of the emergence of universal access to information and the transition from paper to digitally based medical libraries from 1995 to 2015. Four factors had emerged by the mid-1990s which together drove this transition. The first was the emergence of the Internet into academic and research institutions in the early 1990s, the first emergence of the World Wide Web (WEB) in 1992, and the launch of the first widespread browser, Netscape, in late 1994. The Internet and the WEB provided the platform for the widespread networking envisioned and nurtured by NLM in its Integrated Advanced Information Management Systems (IAIMS) Awards program in the 1980s. Second, was the emergence of the Web which enabled NLM to rapidly expand its outreach efforts to facilitate direct user access to KBI at the clinical, educational, and end user levels. Third, in 1994/1995, was the shifting in accreditation by the Joint Commission for Accreditation of Healthcare Organizations (now named The Joint Commission) and other hospital accreditation bodies from a departmental focus to a functional focus.

This change combined with the ability of the Internet and the WEB to greatly facilitate institutional access to KBI focused commercial products such as UpToDate created a major challenge for hospital libraries and their role in the emerging online health information digital world. And fourth, over the next twenty years, as the transition of libraries from being print based to digitally based occurred, the healthcare marketplace changed as the traditional focus of the market on books and journals evolved into a focus on creating collections and new tools to provide more direct and facilitated access to the actual knowledge needed.

In 1995, building on MLAs educational policy statement, NLM's Board of Regents published the report of its Planning Panel of Education and Training of Health Science Librarians. The NLM's 1995 Annual Report lists this report's goals and recommendations to address the need to:

- Prepare for the new forms of information, new users, and new practice patterns that may be required for health sciences librarianship,
- to Match the capabilities of health sciences librarians to the needs of employers,
- to Update and enhance the curricula of schools of library and information science,
- to Foster educational programs enabling health sciences librarians already in the workplace to update and extend their professional education and training,
- to Experiment with alternative methods and courses of study for adult learning,
- to Attract the best and brightest candidates the current market can provide, and
- to Achieve greater cultural and ethnic diversity in the profession.

This report helped prepare NLM and MLA to take advantage of the emerging World Wide Web (WEB) and the transition from a print to a digital KBI/information/data environment which continued for another 20 years until the transition was completed.

This transition from print to digital from 1995 to 2015 occurred in three phases. The Association of Academic Health Science Libraries' (AAHSL) Annual Statistics clearly show the three phases.

### Presenting a Graph on the Shift from Print to Digital: Percent for Electronic Resources

The first phase started in 1995, when the rapid expansion of the World Wide Web began. That year AAHSL's Annual Statistics first listed the percentage of the libraries acquisition budget used for electronic materials. It represented 5.75% of that budget. It consistently continued to grow reaching 16.5% in 2000/01. In this period medical libraries focused on developing websites to provide access to this new category of KBI resources. Library staff had to learn how to not only provide access to and use of these digitally based resources but to be able to authenticate users that their contractual arrangement with their vendors required. As digital collections continued to grow in importance, the period of the transition increased rather than reduced library costs as libraries had to run parallel systems for processing and maintaining print collections and digital resources [1].

**Figure 1:**
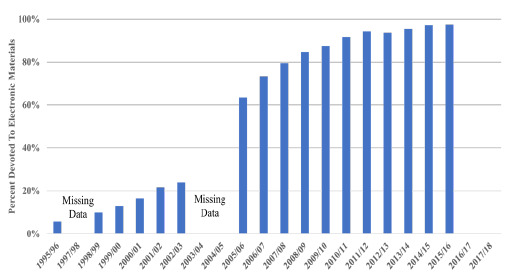
Transition Data Print to Digital: Percent of Collection Budget Devoted to Electronic Materials

By 2005/06, the percentage spent on digital resources had grown to almost 64% and to almost 92% in 2010/11. Medical libraries were able to shift their focus to their digital resources and they were able to reduce the processing needed for their print collections. As part of this shift the role of cataloguers evolved into metadata and electronic resource librarians. From then on the percentage stayed near or above 95% and AAHSL ceased collecting this statistic after 2015/16 as there was so little still being sent on print that this statistic was no longer useful.

### Transition Data Print to Digital

**Figure 2:**
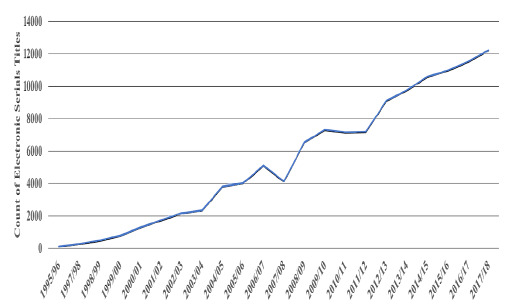
Count of Electronic Serials Titles

This shift of data print to digital was also indicated by the increase in the number of electronic serials titles reaching over 12,000 by 2017/18.

**Figure 3:**
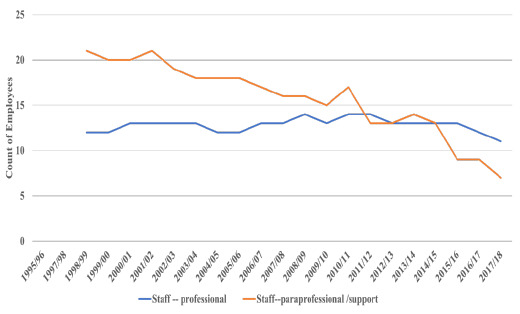
Staff and Professional Employees

With completion of the transition to digital and the decline in the physical processing of print materials the decline in the number of paraprofessional and clerical staff AHSLs employed continued from a high of 21 in1998/99 and 2001/02 to 17 in 2010/11 to 9 in 2015/16 and to 7 in 2017/18.

**Figure 4:**
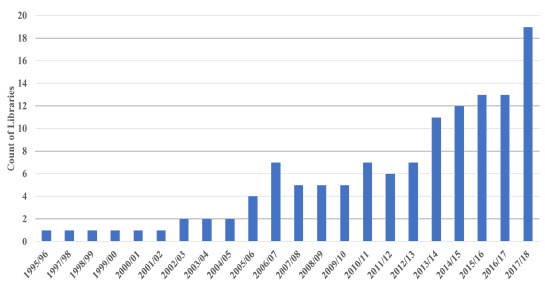
Total Number of Libraries with Fewer than 15,000 Print Volumes

Another indication of the shift was the increase in the number of digitally based libraries with the number of AAHSL libraries with fewer than 15,000 print volumes growing from 4 in 2005/06 to 11 in 2013/14 to 19 in 2017/18. This partially reflected the fact that libraries being developed in new medical schools were primarily digital.

In his 2011 Doe Lecture titled *Breaking the barriers of time and space: the dawning of the great age of librarians,* T. Scott Plutchak discussed the emerging shift from print-based to digital-based libraries and the resulting shift from the focus on the libraries role as the institutional repository of KBI/Information to, in the digital era, a focus on medical librarians/HIPs stating:

“The Great Age of Librarians.” I believe that we are at the threshold. But just at the very threshold—the very beginning. The incunabula period of the digital age [2].

He also states:

In the digital age, physical libraries are becoming less relevant to the communities that they serve. Librarians, however, are more necessary than ever in helping members of their communities navigate the increasingly complex information space. To meet their social responsibilities requires that librarians seek new roles and recognize that their most important activities will take place outside of the physical library [3].

He also pointed out:

It is important to remember that we are just now in the very, very early stages of this transformation from a world of knowledge encoded in print to a world where digital information is the dominant medium, and instantaneous broad communication has radically transformed the ways in which people interact. We do not have established norms and protocols. We are making it up as we go along. Which experiments will bear fruit and which will fall by the wayside remains to be seen. It is unlikely that any of us here will live long enough to see the emergence of a truly mature digital culture that parallels the print culture that we all grew up in… In a world in which intellectual content was encased in physical objects, we built the great library systems of the twentieth century. In the period of the digital world, what should we do instead? [4]

However, partly due to the challenges health science libraries were facing in this transition, as already discussed, and the lack of a clear vision of the emerging digital health related information/data environment the emergence of the new roles of medical librarians in their transition to HIPs did not make significant progress until the transition was completed in the mid 2010's. In the remainder of my talk, I will focus on the emerging digital and data driven information environment and the opportunities and the challenges it presents our profession. I only have time to present an overview of this, but hope to reinforce your understanding that the digital health information environment is much more than simply an online version of our previously print based information base.

### NLM 2017-2027 Strategic Plan Cover

As it was in the 1960's and 70's with its development of the MEDLARS/Medline system and the national medical library infrastructure, NLM has been a principle force in the evolution and advancement of the emerging digital health information environment. Its 2017-2027 Strategic Plan is focused on the development in the digital environment of a new, data driven health information ecosystem. This plan is providing the road map for NLM's lead in creating the new, data driven digital health information ecosystem in much the same way as its creation of the MEDLARS/MEDLINE systems and the implementation of the Medical Library Assistance Act did over fifty years earlier. I will now be quoting from the plan as I think it is important for medical librarians/HIPs to understand the vision and goals of the plan and for them to be following the emergence of the new health information ecosystem it is driving.

The introduction to the Strategic Plan presents an overview of the emerging health information ecosystem it envisions. The introduction to the plan states:

NLM's vision for the coming decade is to unleash the potential of data and information to accelerate and transform discovery and improve health and health care. Central to this vision is the idea of data-powered health, in which holistic knowledge of the person is enriched with greater awareness of biological processes, environmental exposures, and human connection. With data-powered health, extant data complements experimental and observational approaches, care processes are characterized by high levels of personalization, and personal data collection becomes a foundation of self-knowledge and personal health management [5].

While NLM's mission does not mention libraries, the increasing complexity and potential impact of the emerging digital KBI/Information/Data environment will require medical librarians/HIPs to support and collaborate with the health related clinical, educational and research communities they serve. In much the same way that medical librarians saw their status and level of service greatly increase in what in our history we referred to as “The Medline Era – The Golden Age of Medical Libraries”, I believe that, as Plutchak stated, “Librarians, however, are more necessary than ever in helping members of their communities navigate the increasingly complex information space.” We, as a profession, are potentially on the verge of a new “Golden Age.”

### Strategic Goal

The plan presents three goals to advance its achievement of this mission with four objectives for each goal. While all the goals and their objectives have potentially significant impacts on the KBI/information/data environment, five of the 12 objectives should have a direct impact on health science libraries and their HIPs' services and impact. I will briefly review these five objectives including progress in achieving them made over the past 5 years as this will give us a preview of the emerging digital KBI/information/data environment.

The Strategic Plan's first goal's first objective starts with this introduction giving a brief overview of the need for the development of data-driven health-related discovery tools and concepts in the digital information environment:

In the not-so-distant past, a given scientific investigation could be broken down into three interconnected research products: (1) the data collected during the course of experiments; (2) the tools and software used to draw conclusions from the data; (3) the resulting publication that reports conclusions drawn from the data and analysis.

This references the paper era.

Today these research products are largely digital, as are other products and processes, including models and simulations, data-standards, workflows, and more. … An emerging digital environment — including new methods, more openness, and greater computational and storage capacity — provides the opportunity to link the various processes and products of science into an interconnected ecosystem of digital research objects, bringing a multi-dimensional view of the research endeavor into full relief.

The overview of how NLM will work over the next ten years to support the development of this new digital environment is presented next:

NLM aims to support innovation that explores alternate paths of re-analysis and discovery through dynamic visualizations and executable articles. NLM intends to create computable libraries of data, models, literature, and more to devise an interlocked environment that enables and enhances discovery and stimulates insight [5].

Next it gives a brief description of the new digitally based information ecosystem:

In the process, NLM will leverage its leadership to advocate for research practices and policies that foster open science and scholarship. NLM will link its expanding collections into an ecosystem that ensures digital research objects are FAIR (findable, accessible, interoperable, and reusable). Facilitating data reuse will work to enhance reproducibility and rigor, a central priority for NIH-supported research. And, ensuring discoverability while democratizing access can underscore the interconnections between research outputs throughout the course of scientific inquiry [5].

Goal One's second objective is supporting research in biomedical informatics and data science, “in close partnerships with the biomedical and clinical research disciplines,” to build the discovery and knowledge resources and tools needed to be able to integrate and use the new types of data in the new digital health information ecosystem presented in Objective 1.1.

Below are examples presented in the Strategic Plan of research and resource development needed to achieve the effective data-driven health research and clinical capabilities to effectively achieve the potential positive impact of the emerging ecosystem on the nation's health:

With the science and health sectors producing more digital research objects—whether research-generated datasets, electronic health records, or computational models—value can be added through curation. A key area for research is how curation at scale might be accomplished for hundreds of millions of such objects in a way that would be workable across biomedical domains, healthcare sectors, public health, and consumer health [6].

HIPs will need to work with health informatics related experts in developing the platforms and the organization of their content to facilitate efficient access and use of these vast health related data resources. HIPs will also need to become experts in the effective access and use of these resources for clinical care, research, and institutional planning and evaluation.

- Analytics beyond statistics, data presentation beyond visualization, and “data mining using meaning” across heterogeneous data sources are important areas for advancing data science. Research and development of artificial intelligence in its many forms, including natural language processing and deep learning, require stimulation, demonstration, testing, and curation.
- Computable biomedical knowledge—such as diagnostic, predictive, and decision-analytic models, or practice guidelines represented as coded digital objects—is an increasingly important complement to human-readable knowledge represented in books and journals.
- Define the structure and functions of “executable articles” that form an interactive library where the resources “talk to each other” and enable movement from data to knowledge and knowledge to action.
- A rich set of data resources presents the opportunity to pursue basic research on data-driven questions in biomedical domains. NLM will partner with scientists across NIH to generate research questions and identify value solutions.

HIPs will need to understand how to use the new tools being developed and their underlying structure to support the value and use of this expanded data environment.

## EMERGING CONCEPTS AND INNOVATIONS IN THE EMERGING ERA OF COMPUTABLE BIOMEDICAL KNOWLEDGE (CBK)

Since 2017, significant progress has been made accomplishing this goal. I am now going to briefly review emerging concepts and innovations in the emerging era of computable biomedical knowledge (CBK):

1. **Computable Biomedical Knowledge (CBK)**
  The Manifesto of the Mobilizing Computable Biomedical Knowledge (MCBK) community states; “Computable biomedical knowledge (CBK) is the result of an analytic and/or deliberative process about human health, or affecting human health, that is explicit, and therefore can be represented and reasoned upon using logic, formal standards, and mathematical approaches” further stating “This should be done using computable formats that can be shared and integrated into health information systems and applications.”
2. **Common Data Elements (CDEs)**
  CDE's are “structured human and machine-readable definitions of data elements that have been recommended or required by NIH Institutes and Centers and other organizations for use in research and for other purposes.” In 2015 NIH launched the NIH Common Data Elements (CDE) Repository creating a national database of common data elements which NIH encourages researchers within the various areas of health and disease research use to strengthen the accuracy, consistency and interoperability of their data and their results [7].
3. **The Resources for Data Driven Discovery (RD3) Portal**
  Introduced by the National Network of Libraries of Medicine in 2017. It “fosters learning and collaboration in data science and data management”. The portal primarily serves librarians and library administrators working in institutions that are generating, sharing, storing, and/or using data for basic scientific, clinical, or translational research and teaching in the health, biological, and physical sciences. It brings together resources on education, outreach and collaboration, best practices, and current events for data-driven research. The portal provides librarians with the tools, knowledge, and skills to effectively participate in networked science and teach data topics to the Network of the National Library of Medicine (NNLM) stakeholders.
4. **FAIR Guiding Principles for scientific data management and stewardship**
  A guiding principle of the NIH Strategic Plan for Data Science, and the approach of the Office of Data Science Strategy (ODSS), is that all biomedical research data should adhere to FAIR principles. This means data should be findable, accessible, interoperable, and reusable (FAIR). While there is no one-size-fits-all solution, ODSS advocates for alignment to the FAIR principles across the biomedical research enterprise [8].
5. **COAR – Confederation of Open Access Repositories**
  COAR grew out of a project in the last years of the 2000s to connect European repositories. Its Mission is “To enhance the visibility and application of research outputs through collaboration across the global repository network” [9]. It has now grown to 155 members with members in six continents. Taking a major role in repository development and management was an early collaborative role for HIPs.
6. **Learning Health Systems**
  The *Agency for Healthcare Research and Quality's* (*AHRQ*) defines a learning health system as “a health system in which internal data and experience are systematically integrated with external evidence, and that knowledge is put into practice. As a result, patients get higher quality, safer, more efficient care, and health care delivery organizations become better places to work” [10]. This approach often provides the framework for systems built upon data of different types integrated with the above concepts to achieve specific clinical, research, educational, or managerial objectives.

The above information types will be able to be integrated by enhancing their data to turn it into computable biomedical knowledge (CBK) to make the data consistent and compatible via their translation into Common Data Elements (CDEs).

As noted earlier we are in the very early evolution of the digitally based health information environment. The above tools, concepts, and resources are still being developed but are emerging as standards and tools. In an email response to a query from me on types of data, Gerald (Jerry) J. Perry, Associate Dean, University Libraries, University of Arizona and the 2019 Doe Lecturer stated that in addition to traditional forms of data:

An emerging format that you may also want to consider is not just data but computable information, such as code or machine-readable and executable commands. A group I have been involved with is the Learning Health Systems folks at the University of Michigan School of Medicine, who are focusing on this, and we refer to this content as computable biomedical knowledge (CBK). An example I like to use is where a piece of code is embedded into something like an electronic patient record. When that code executes, it locates and acts on information or data and generates knowledge that can then be acted upon. These CBK elements need curation and management as assets, just like data (and they often use data), but they are in and of themselves not necessarily data. Artificial intelligence agents, machine readable artifacts, algorithms - those sorts of things [11].

## LEARNING HEALTH SYSTEM IN SUPPORT OF A HOSPITAL

The above emerging data driven information environment sounds impressive but I initially had trouble envisioning what it would look like. So I envisioned an example of a possible clinical Learning Health System in support of a hospital patient's clinical care and monitoring. It could be the development of a system which integrates:

- Patient information including their medical and medication history, physical information and confounding conditions from his or her electronic medical record;
- Data from active clinical monitoring devices;
- Evidence-based information relating to the patient's clinical condition, treatment options, and personal health related preferences.

Using Artificial intelligence agents, machine readable artifacts, algorithms and new analytical tools as they are developed, the system would provide the clinical team a much more developed and effective clinical care support resource to monitor and guide the specific patients care and treatment.

**Figure 5:**
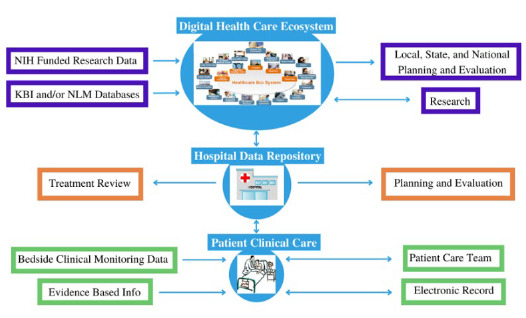
Learning Health System in Support of a Hospital Patient's Clinical Care and Monitoring

The information created would then be de-identified and accumulated in the hospital's local data repository. These ‘data' can then be used by the hospital for treatment review, and for planning and accreditation support.

Researchers (internal or external) can then combine these data with comparable data from other clinical or research-oriented institutions as well as data collected in NIH funded research, to research clinical questions such as clinical treatments and how they are affected by the patient's physical or medical history, medication and treatment effectiveness, disease etiology and impact correlated with their demographic characteristics and so forth. Aggregated data could also be used on the local, state, and national level for planning and evaluation purposes.

The development of such systems and the interfaces to facilitate their access in use in various settings will not be simple and the need for collaboration and support of health information experts in all phases of the development, operation and use of such systems is crucial. A current major focus of NLM is the development of the AI based tools to facilitate the curation of the data in the various phases of a learning health system such as I have postulated. Medical librarians/HIPs have the understanding of health-related literature and research and the skills in their organization and have extensive experience in supporting their access and use. Here lies the potential for our profession to enter a new golden age but we must develop the vision, skill sets and knowledge HIPs will need if we are to achieve Plutchak's “great age of librarians.” Medical librarians/HIPs are needed more than ever in helping members of their communities navigate the increasingly complex digital health information ecosystem. Meeting this information need will require that they upgrade their knowledge and technical skills to enable them to serve in new collaborative roles in health-related research, clinical and professional education, and recognize that their most important activities will take place outside of the physical library [12].

Current medical librarians/HIPs need to be familiar with these concepts and tools and with their significance in the new emerging health information ecosystem. I have provided a listing of these with their URLs for further access. The new roles and skills needed by Health Information Professionals in this era will be discussed in my next section.

NLM's Strategic Plan Goal one's third objective states:

NLM will strongly support and advocate for open source policies and objectives to provide access to the products and processes of scientific research to make it widely accessible [13].

This objective focuses on the need to make potential health related data, including clinical data, available for use for clinical and research activities while protecting patient specific information and the copyright protection of the data's producer. It focuses on the continued advancement of the open source movement which health science libraries and HIP's have been advocating for and supportive of. NIH's requirement for projects funded by NIH that required open access to resulting publications be made accessible with in twelve months of publication was an initial requirement for this objective. On January 25, 2023 a new Final NIH Policy for Data Management and Sharing replaces the previous policy established in 2003. The new policy requires NIH funded researchers to prospectively submit a plan outlining how scientific data from their research will be managed and shared [14].

Medical librarians/HIPs should play a key role as the experts on the access and use of the new research information types and systems being developed such as open science including access to new research data sources.

Goal 2 focuses on enhancing “dissemination and engagement pathways”. This is a continuation of NLM's focus since 1991 on facilitating direct access to health related information to meet the needs of health related research, clinical care and the general public including underserved communities. Health Science Libraries and HIPs need to expand their role in facilitating and supporting end user access and use of the expanding digital KBI/Information/Database.

NLM is drawing upon its recently renamed Network of the National Library of Medicine for much of the research and implementation of tools and programs to support this expanded access to the expanded digital health ecosystem. This will provide opportunities for medical libraries and their HIP's to directly contribute to the development and access to the new “dissemination and engagement pathways” being developed. It is important that Health Science libraries and HIPs understand, coordinate and exploit NLM's efforts in this area in serving their institutional community including how their patrons serve their client populations.

Goal three - Developing the Librarian Workforce for Data Science and Open Science is also critical in the evolution of the services of health science libraries and HIPs in this new era. HIPs will need to develop their biomedical and technical training and skills on the access, management and use of the rapidly expanding health information ecosystem with its focus on data driven discovery.

As the introduction of Goal 3 states:

To assure a future of data-driven discovery and health, it is essential to have a biomedical informatics and data science workforce prepared to make conceptual and methodological advances in analytics, visualization, mining, and other methods needed to use data for discoveries and to make it interoperable with existing knowledge. A new generation of librarians who are as capable of collecting and disseminating data resources as they are with more familiar forms of scholarly communications is also needed. The roles of the informationist and the librarian must expand to support a world where massive industrial search engines provide immediate access to vast stores of knowledge, while threatening to overwhelm the scholar, patient, and clinician with unfettered lists of potentially relevant resources [15].

A review of the advances in preparing HIP's for their new roles in the digital environment needs to be made in the context of a review of the emerging scope and nature of these new roles. The next section with review these together.

NLM and MLA have long provided training and support for medical librarians. Since 1946, when MLA shifted from an organization of medical libraries to an organization of medical librarians, it has supported the upgrade of medical librarianship from a vocation to a profession. Its launching of its certification program in 1949 was seen as a major step in this. While MLA's support in the professional and technological support and training of its members would be a good topic for a future Doe Lecture I want to review its more recent activities in support of medical librarians/HIPs in the shift from the paper to the digital health information environment.

MLA in the decade of the 1980s called for a reevaluation of the educational needs of library students by their graduate programs and for MLA to work with the graduate programs to develop curricula to prepare their graduates with the essential skills and knowledge in the new technologies. MLA's 1987 strategic plan further recognized the need to define the knowledge and skills needed by medical librarians. This was formally recognized in 1988 with MLA's transition of its accreditation program launched in 1949 to the Academy of Health Information Professionals (AHIP).

In 2000 Davidoff and Florence published in an Annals of Internal Medicine [16] editorial a call for the creation of a new profession they called an informationist to be added to the clinical care team to better locate and integrate KBI into their patient care. However, they did not see the hospital librarian filling this role calling for the informationist to be someone with both library and medical training. This is an early example of our profession's need to upgrade the training and services of our medical librarians/HIPs.

In 2007, reflecting the impact of the Internet and the WEB, MLA updated its *Policy for Change* with the release of its *Competencies for Professional Success* report. In 2017, the 2007 report was updated with the release of MLA's *Competencies for Lifelong Learning and Professional Success*. These reports continued the shift of health science librarians from medical librarians to health information professionals. The 2017 Report used the health information professional (HIP) terminology exclusively stating:

We chose “health information professional” as an inclusive label to refer to the professional group for whom these competencies are relevant. “Health information professional” is part of the name of the Academy of Health Information Professionals, and the 2015 MLA strategic plan has as an educational goal to “position MLA as the go-to education resource for health information professionals” [17].

Between the 2007 and the 2017 reports there were several studies reviewing the literature to identify new, emerging roles but the only one getting traction was that of data librarian. After the publication of the 2017 report, the completion of the transition to primarily digital collections, and the launch of NLM's new strategic plan, reviews of new roles using these new developments as a framework for evaluating the emerging roles developed.

The following are two examples of exploration of new roles for medical librarians/HIPs.

## ON MA ET AL.

In a 2018 article published in JMLA titled “Emerging Roles of Health Information Professionals for Library and Information Science Curriculum Development: A scoping review,” Ma et al searched studies which identified emerging and evolving roles filled by HIPs [18]. They presented the specific competencies presented in the 2017 report most relevant to each of the roles identified in the studies they reviewed in the literature (see Appendix A, slide 12).

## RESEARCH CYCLE

As the role of medical librarians/HIPs has shifted from a support role to a collaborative one HIPs have become more involved as part of research teams throughout the research lifecycle. This slide (see Appendix A, slide 13) presents a model of the research lifecycle and the library's role in the different steps of the cycle developed by the University of Central Florida's Library. The Library's HIPs are involved in every step of the process and their roles will continue to grow with the continued development and advancement of Computable Biomedical Knowledge (CBK) based systems.

## MLA DATA SERVICES SPECIALIZATION

Recognizing that data support services was the most widely recognized new role for HIPs, in 2020 MLA launched a new Data Services Competency [19]. The commentary in the April 2020 issue of JMLA announcing the new competency program describes five performance indicators of data management describing the skills to be able to meet the indicators. In January of this year, MLA announced the list of course work required for certification at the first level of this certification with the development of the second level still in process. The courses are presented in a grid structure listing indicating which courses satisfy each competency [20].

Dr. Lisa Federer, the Data Science and Open Science Librarian at NLM who has been an active member of MLA, lead and coordinated both MLA and NLM's strong support of this development. NLM has taken a number of steps to build upon MLA's work in developing the emerging new roles for HIPs in the new digital information ecosystem including:

- Developing the Librarian Data Science and Open Science Workforce held April 15-16, 2019
- Providing training on Biomedical Informatics, Data Science, and Data Management.
- The establishment of the National Center for Data Sciences (https://nnlm.gov/about/centers/ncds) in October 2021.
- NLM Curation at Scale Workshop (March 2022 - https://www.nlm.nih.gov/curationworkshop2022/index.html)

The Association of College and Research Libraries (ACRL) has also been supporting academic librarians in developing skills and services in data management. For example, publishing in 2021 its Scholarly Communication Toolkit: ACRL Workshop: Research Data Management stating:

Research data management has emerged as a need among academic researchers and librarians are building skills in response. This one-day workshop will assist liaisons to identify their existing skills and mindsets that transfer to research data management services and then create a learning plan for the RDM specific knowledge needed to serve their subject disciplines [21].

An important question in the development of new roles of HIP's in the emerging era is that of professional boundary issues between medical library HIPs and health informationists. This issue of professional boundary issues was recognized in 2005 in Perry et al, “A Current Perspective on Medical Informatics and Health Sciences Librarianship” which states and discusses that “boundaries are disappearing among the published literature, research data, research databases and clinical patient data” [22].

Another aspect of the challenges facing medical librarians has been and is the lack of adequate preparation of library students to carry out research. In 2009 McKnight and Hagy reviewed the web sites of the fifty American Library Association accredited Master of Library and Information Science programs. As in an earlier study by Park in 2002 they found that while all but one of the programs include a research methods course about fifty percent of the programs had no indication on their website that such a course was required [23]. They concluded that it is not reasonable to expect all of their graduates to be highly skilled in conducting and publishing research [24].

## CONCLUSION

In summary, the emerging digital health information ecosystem presents health science libraries and their medical librarians/health information professionals with significant challenges and opportunities. We can develop our services and contributions to facilitate and support its development, organization, effective access and use to more effectively impact the improvement of health care in this country or to slowly let our libraries and librarians fade into the background as what we have done in the past fades in importance.

Led by their professional organizations – the Medical Library Association and the Association of Academic Health Sciences Libraries – medical librarians/HIPs need to understand the significance of the transition from the print to the digital health information environment.

The shift of medical librarians to HIPs who have been becoming more closely embedded in the programs and research teams they work with over the last twenty years has represented the start in the shift from their role of support staff to full collaborators. As the data driven health information ecosystem being guided by NLM continues to emerge it is crucial that the skills and services of our HIPs continue to upgrade and develop. I want to close with a brief review of my thoughts on where we are in this shift to HIPs:

- We need to work with health informaticians to integrate published literature, research data, research databases, and clinical patient data in knowledge sources to support clinical decision making and research. HIPs can take a role in the creation, maintenance, and development of these integrated information resources.
- We need to continue to expand our role as part of research teams in the same way biostatisticians are part of research teams. Among the skills and expertise we can bring to the team include:
  - Executing literature reviews, synthesis including systematic reviews, literature scoping, and mapping;
  - Coordinating and supporting access to and management of data;
  - Facilitating access and use of Computable Biomedical Knowledge (CBK) data sources and tools which include:
    1. Data generated from scientific research,
    2. Curation and standards,
    3. Data science tools and other executables,
    4. Clinical data,
    5. Status indicators of the health of people and communities,
    6. Health information for the public;
  - Support Survey tool development and use;
  - Serve as institutional repository developers and managers;
  - Collaborate in the preparation of research grant and other funding proposals;
- We need to upgrade our support of the educational activities of the units and departments we support as instructional design experts and collaborators including:
  - Work with programs and instructors in effective teaching of bioscience, clinical, and health information literacy,
  - Support curricula development using contemporary instructional design principles,
  - Support the use of learner-centered instructional approaches,
  - Teach and support faculty and instructors' use of innovative instructional and communication methods and technologies;
- We need to expand our role as clinical support librarians:
  - Provide instruction in use of KBI/information in support of clinical decision making,
  - Collaborate with clinical care teams
  - Facilitate access and use of Computable Biomedical Knowledge (CBK) data sources and tools in development of systems and tools directly supporting clinical patient care which include:
    1. Data generated from scientific research,
    2. Curation and standards,
    3. Data science tools and other executables,
    4. Clinical data,
    5. Status indicators of the health of people and communities,
    6. Health information for the public.

The above shift from medical libraries to HIPs working in close collaboration with the programs, faculty, instructors, and clinicians they support is crucial in achieving the “Great Age of Medical Librarians” envisioned by Plutchak in 2011.

This is both the challenge and the opportunity you are facing in this emerging new era of our profession. I look forward to watching how you meet this challenge!

## SUPPLEMENTAL FILES

**Appendix A:** Kronenfeld Doe Lecture Powerpoint
