# Supplementary material for: 2022 Janet Doe Lecture, health science libraries in the emerging digital information era: charting the course
Source: J Med Libr Assoc. 2023 Apr 21;111(1-2):555–65. doi: 10.5195/jmla.2023.1626 (PMC10259618; doi:10.5195/jmla.2023.1626)
Supplement: Supplementary file 1 — Appendix A: Kronenfeld Doe Lecture Powerpoint [file jmla-111-1-2-555-s01.pdf]

Internal use

‘Founding Mothers’ of Medical Librarianship

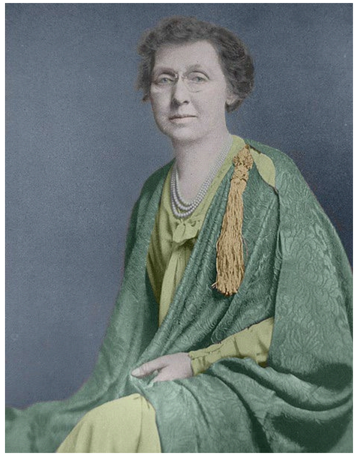

Marcia Noyes

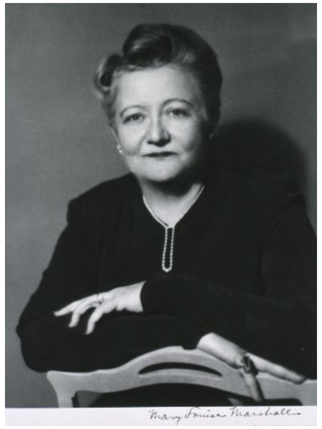

Mary Louise Marshall

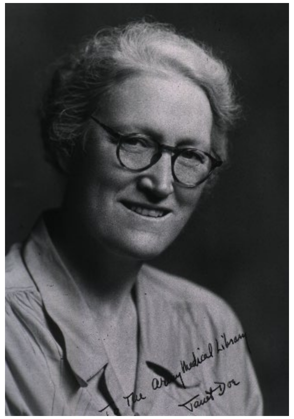

Janet Doe

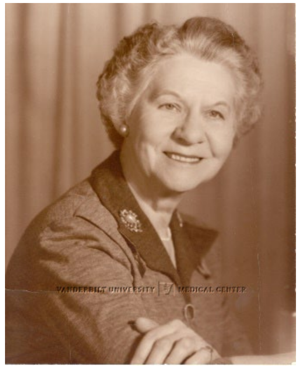

Eileen Cunningham

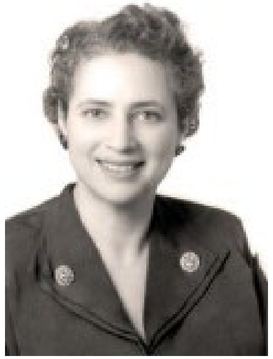

Estelle Brodman

Internal use

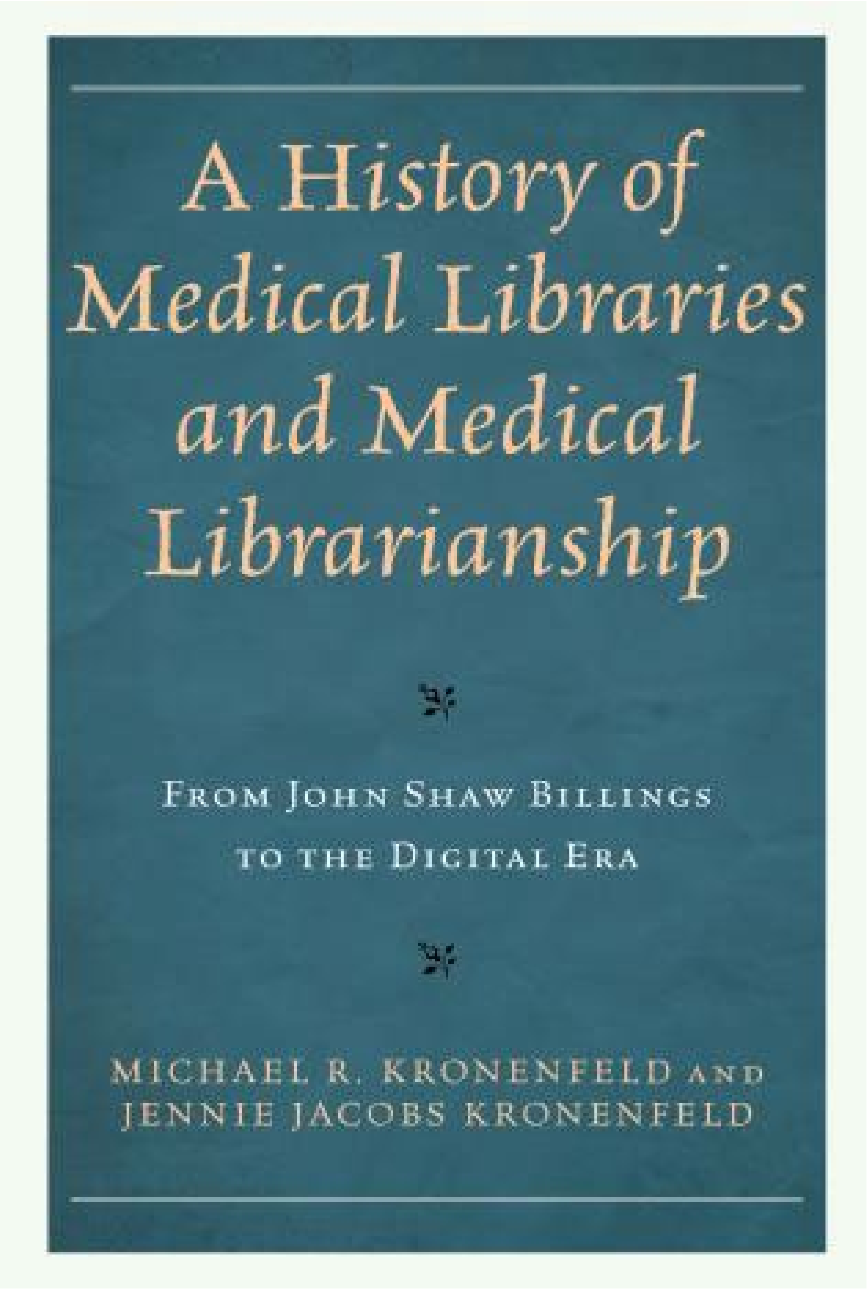

MEDLARS Computers and NNLM Regions

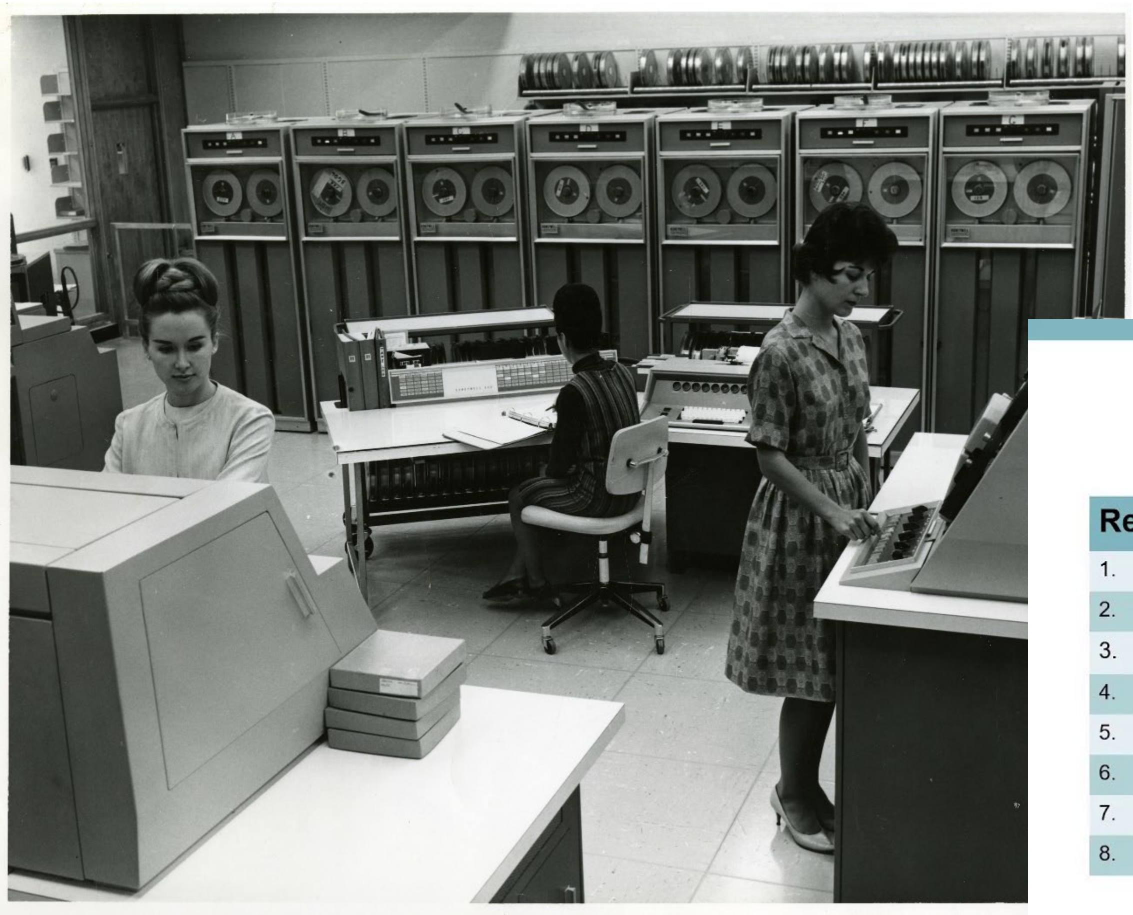

National Network of Libraries of Medicine (NNLM)

nnlm.gov/members/join-network

Regional Medical Libraries		
1.	Middle Atlantic Region (MAR)	Pitt
2.	Southeastern/Atlantic Region (SEA)	UMB
3.	Greater Midwest Region (GMR)	UI
4.	MidContinental Region (MCR)	Utah
5.	South Central Region (SCR)	UNT
6.	Pacific Northwest Region (PNR)	UW
7.	Pacific Southwest Region (PSR)	UCLA
8.	New England Region (NER)	UMass

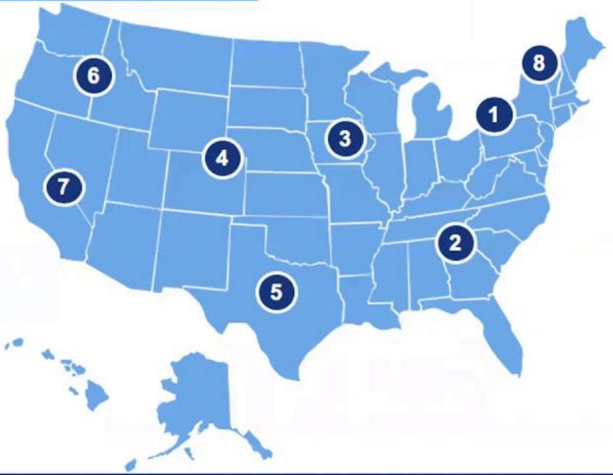

NIH U.S. National Library of Medicine
National Network of Libraries of Medicine

nnlm.gov

Transition Data - Print to Digital – Percent of Collection Budget Devoted To Electronic Materials

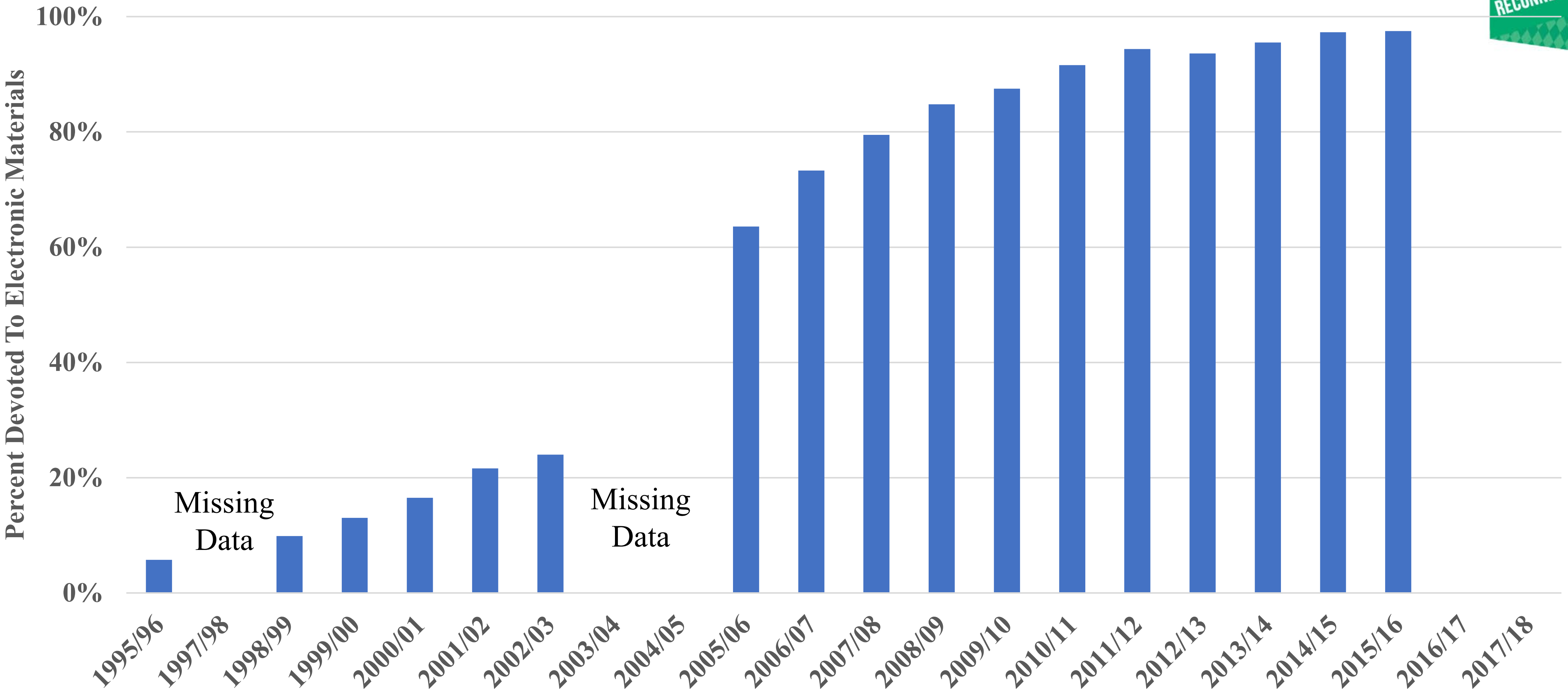

MLA '22 NEW ORLEANS MAY 3-6 2022
#MLANET22
MLANET.ORG
RECONNECT, RENEW, REFLECT

MLA '22 NEW ORLEANS MAY 3-6 2022
#MLANET22
MLANET.ORG
RECONNECT, RENEW, REFLECT

Count of Electronic Serials Titles

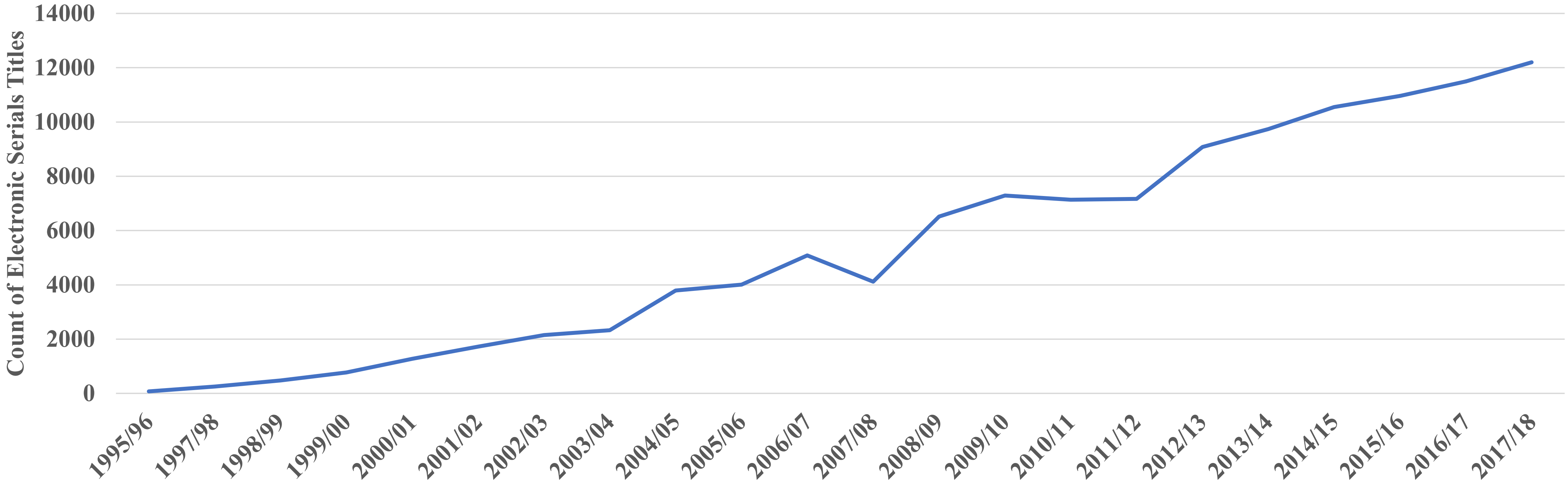

Transition Data Print to Digital – Staff and Professional Employees

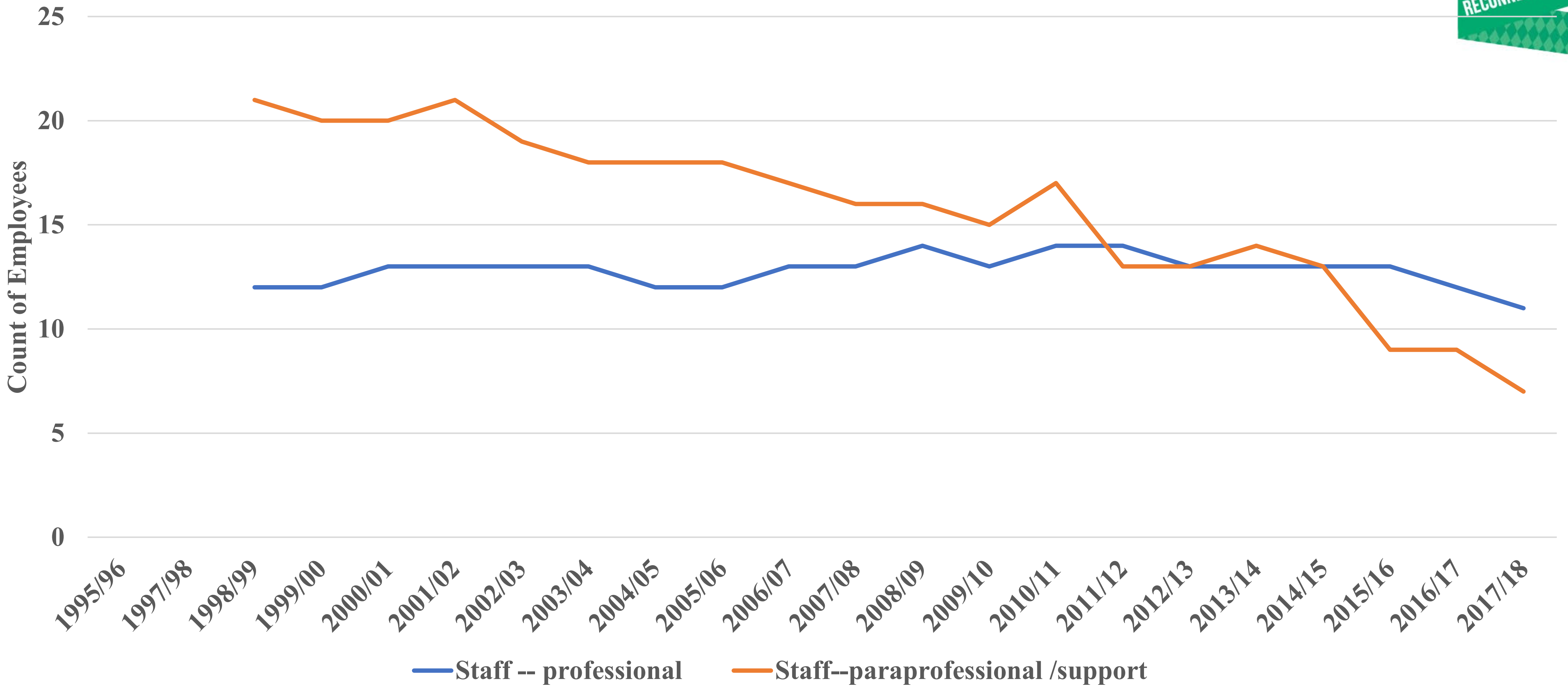

Internal use

Transition - Data Print to Digital – Total Number of Libraries with Fewer than 15,000 Print Volumes

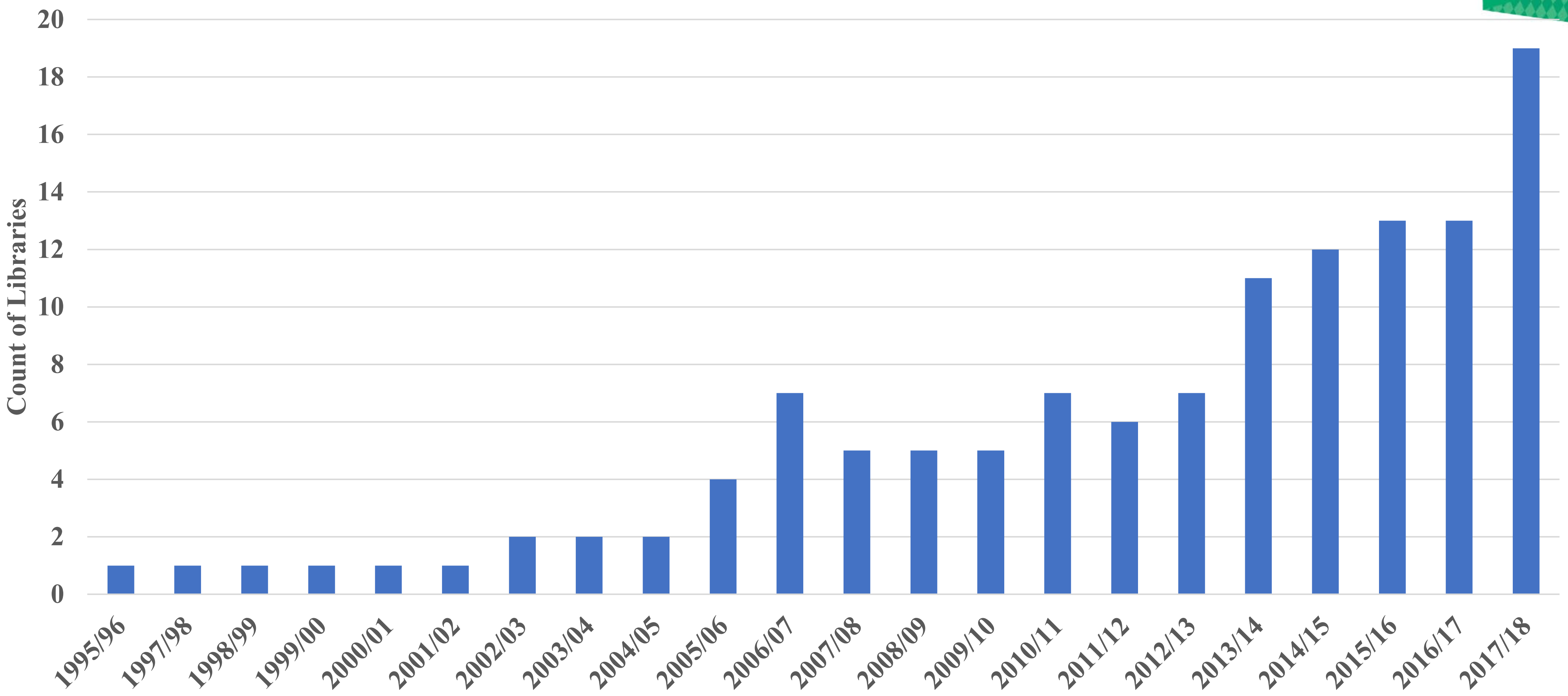

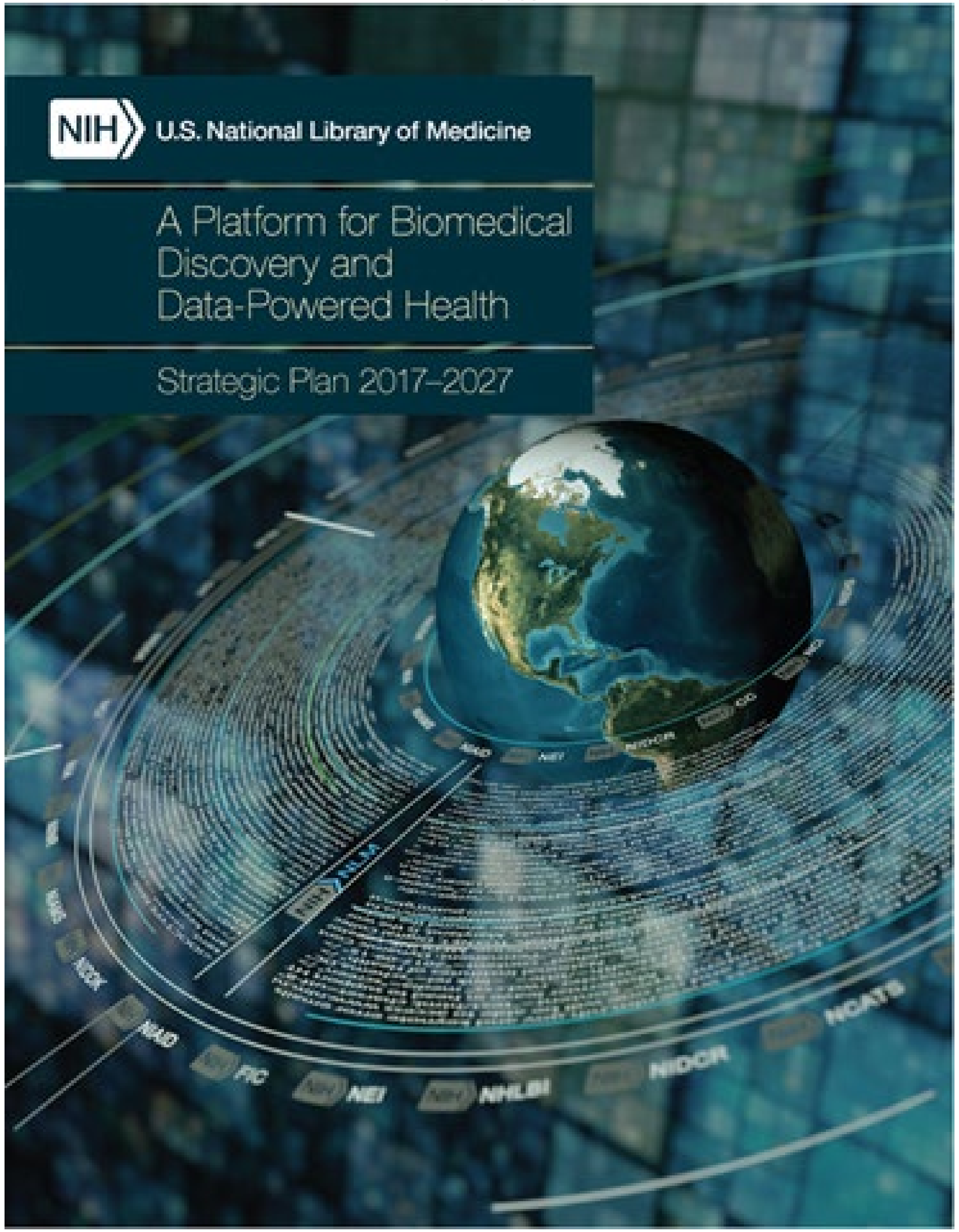

MLA '22 NEW ORLEANS MAY 3-6 2022
#MLANET22
MLANET.ORG
RECONNECT, RENEW, REFLECT

NLM 2017-2017 Strategic Plan Goals

GOAL 1

Accelerate discovery and advance health through data-driven research

1. **Connect the resources of a digital research enterprise**
2. **Advance Research and development in biomedical informatics and data science**
3. **Foster open science policies and practices**
4. Create a sustainable institutional, physical, and computational infrastructure

GOAL 2

Reach more people in more ways through enhanced dissemination and engagement

2.1 Know NLM users and engage with persistence

2.2 Foster distinctiveness of NLM as a reliable, trustable-source of health information and biomedical data

2.3 Support research in biomedical and health information access methods and information dissemination strategies

2.4 Enhance information delivery

GOAL 3

Build a workforce for data-driven research and health

3.1 Expand and enhance research training for biomedical informatics and data science

3.2 Assure data science and open science proficiency

3.3 Increase workforce diversity

3.4 Engage the next generation and promote literacy

Internal use

Emerging concepts and innovations in the emerging era of computable biomedical knowledge (CBK):

- **Computable Biomedical Knowledge (CBK)**
- **Common Data Elements (CDEs)**
- **Resources for Data Driven Discovery (RD3) Portal**
- **FAIR Guiding Principles for scientific data management and stewardship**
- **COAR – Confederation of Open Access Repositories**
- **Learning Health Systems**

Internal use

Learning Health System in Support of a Hospital Patient's Clinical Care and Monitoring

MLA '22 NEW ORLEANS MAY 3-6 2022
#MLANET22 MLANET.ORG
RECONNECT, RENEW, REFLECT

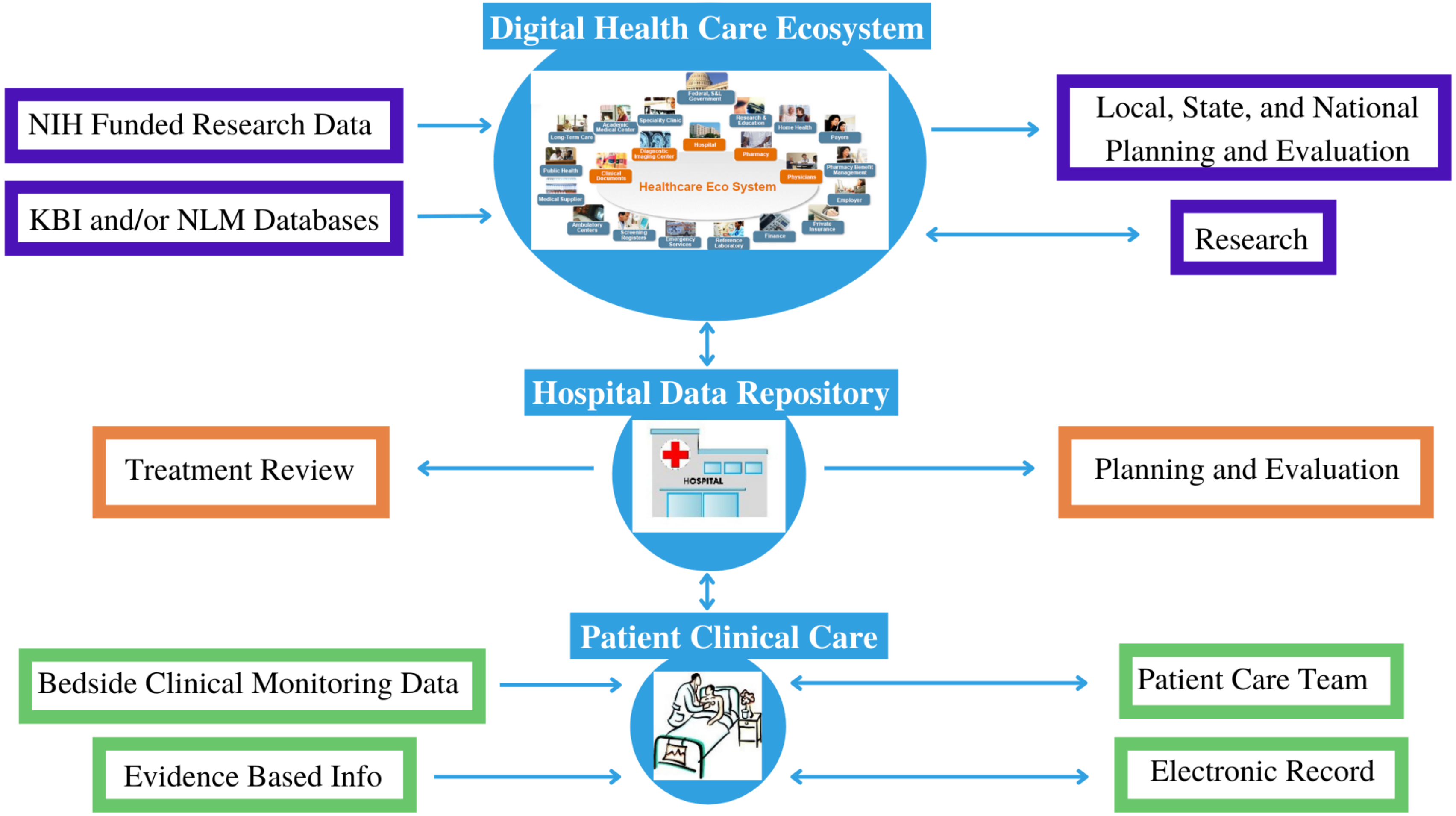

Ma, Jinxuan et al. "Emerging roles of health information professionals for library and information science curriculum development: a scoping review." *Journal of the Medical Library Association: JMLA* 106, 4 (2018): 432-444.

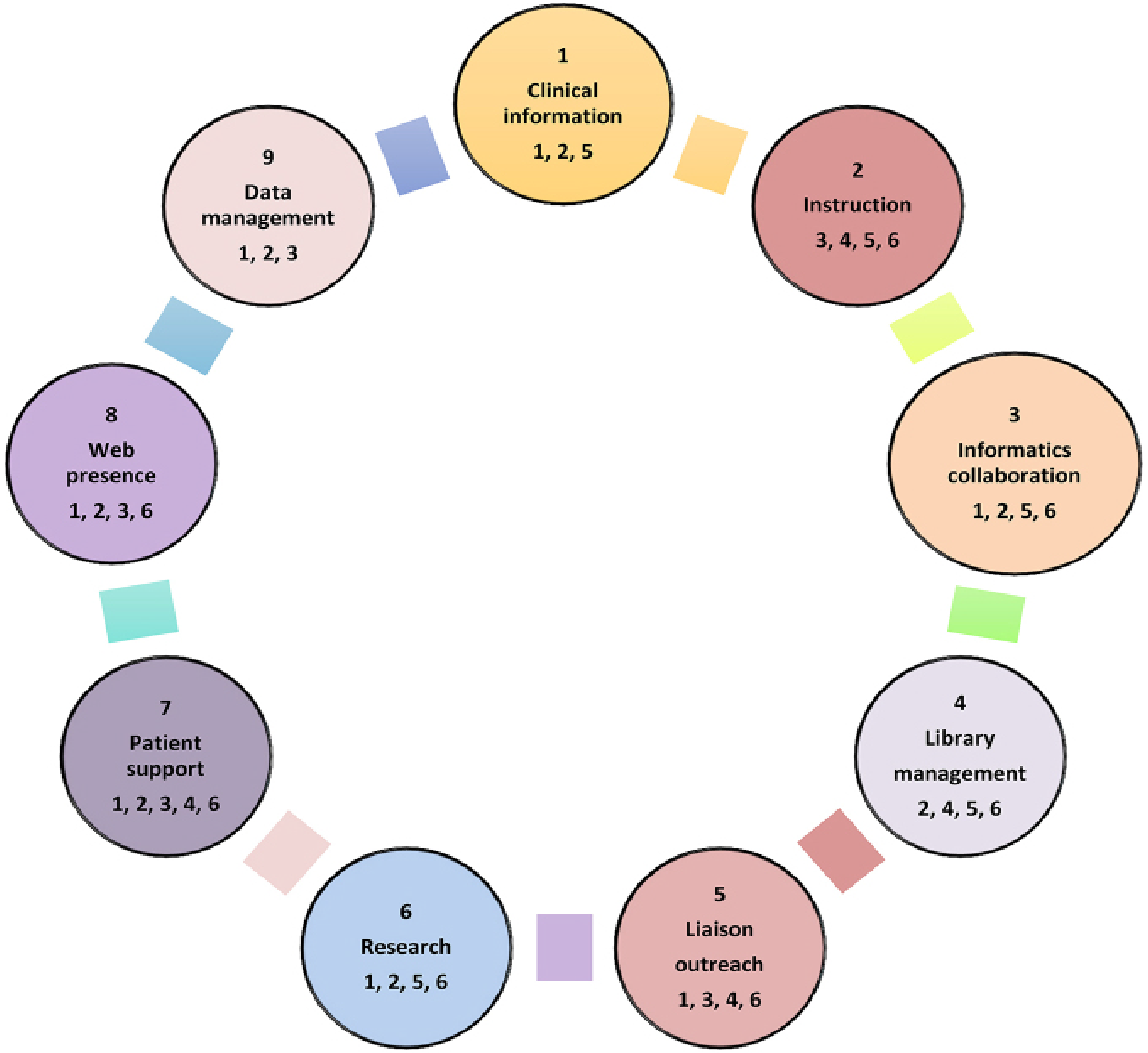

University of Central Florida Research Lifecycle of Research Support and Services

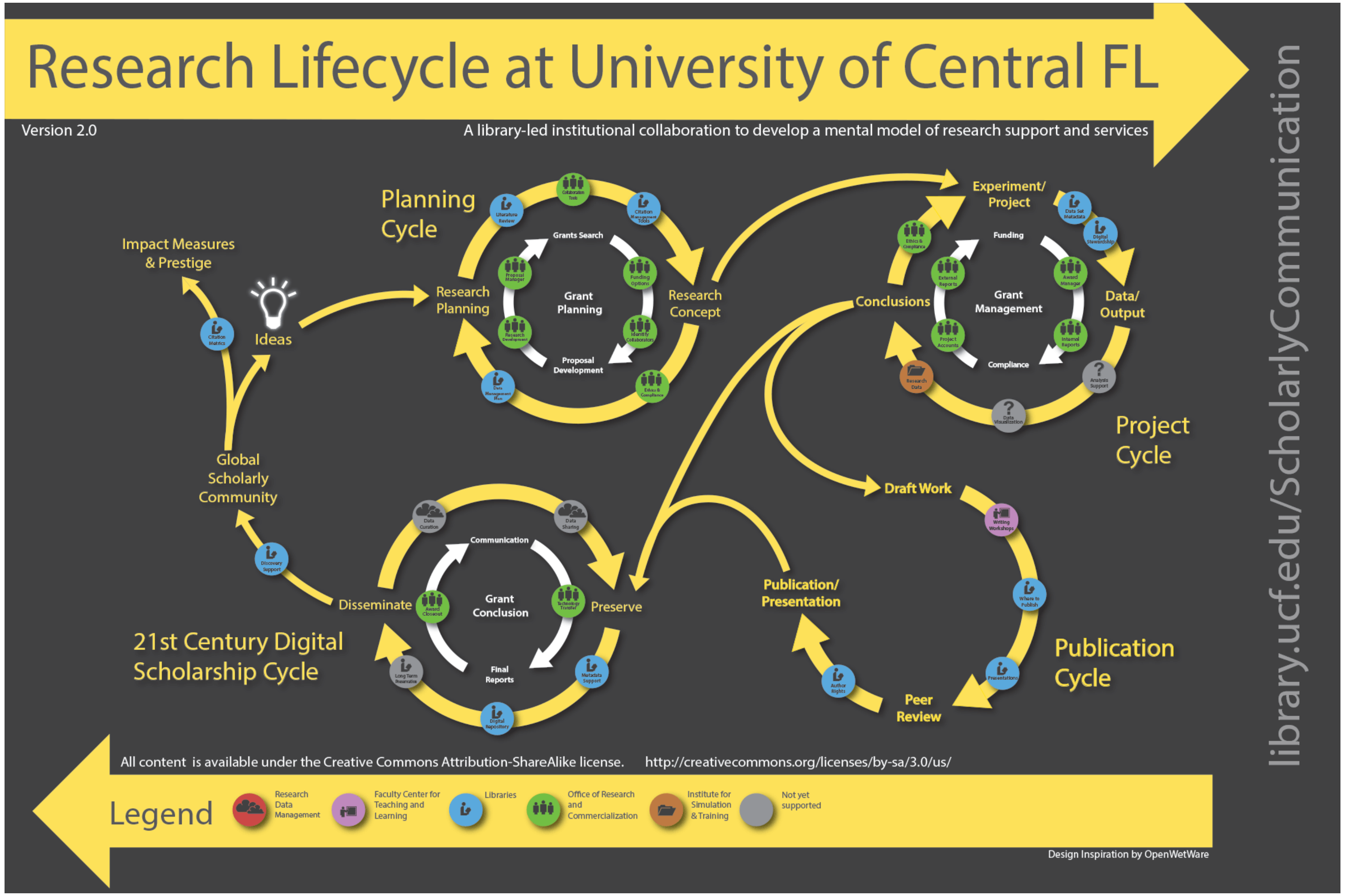

MLA '22 MAY 3-6 2022 NEW ORLEANS #MLANET22 MLANET.ORG RECONNECT, RENEW, REFLECT

Internal use

MLA Data Services Specialization

Earning the MLA Data Services Specialization (DSS) indicates that you have received training in providing data services to researchers, clinicians, students, librarians, and others who work with data--and recognition for the accomplishment of acquiring skills in providing data services. MLA offers a Level I DSS certificate. A Level II DSS certificate is planned for 2022.
